# Work stressors, work-family conflict, parents’ depressive symptoms and perceived parental concern for their children’s mental health during COVID-19 in Canada: a cross-sectional analysis

**DOI:** 10.1186/s12889-023-17037-0

**Published:** 2023-11-07

**Authors:** Jaunathan Bilodeau, Amélie Quesnel-Vallée, Thomas Poder

**Affiliations:** 1 Department of Sociology, McGill University, 3460 McTavish Street, Montreal, QC H3A 0E6 Canada; 2 Department of Epidemiology, Biostatistics and Occupational Health, McGill University, Montreal, Canada; 3 École de Santé Publique, Université de Montréal, Montreal, Canada

**Keywords:** Children, Depressive symptoms, Mental health, Teleworking, Work-family conflict, Work stressors

## Abstract

**Background:**

Work-related stressors and work-family conflict are important social determinants of mental health. While the impact of these stressors on parents’ mental health is well documented, we know comparatively less about their impact on children’s mental health. Furthermore, though the COVID-19 pandemic has significantly altered these stressors, particularly with the increase in teleworking, major knowledge gaps persist regarding the association between parents’ stressors and perceived parental concern for their children’s mental health during the COVID-19 pandemic. Based on the stress contagion perspective, this study tests (1) the mediating role of parents’ depressive symptoms with parental concern for their children’s mental health, and (2) whether these associations vary depending on whether parents had the opportunity to engage in telework.

**Methods:**

A path analysis was performed from a cross-sectional analytic sample of 780 employed parents in the province of Quebec (Canada). The same model was then stratified by teleworking opportunity. The model’s indirect associations were obtained by the bootstrap bias-corrected method with 1,000 replications.

**Results:**

The results show that the stressors of work-to-family conflict, increased difficulties in work-family balance since the COVID-19 pandemic, irregular schedules, low esteem derived from work, and job insecurity were all indirectly associated with an increase in parental concern for their children’s mental health through increased parents’ depressive symptoms. However, some associations differ depending on teleworking status. The indirect associations involving increased difficulties in work-family balance since the COVID-19 pandemic as well as irregular work schedules were observed only in the teleworking group.

**Conclusions:**

This study fills a gap in research on the association between the work-family interface and parental concern for their children’s mental health during the COVID-19 pandemic. It highlights the importance of concerted and cohesive action between child health policies and those regarding work and work-family balance to prevent work-related psychosocial risks, particularly considering the post pandemic expanded and persistent reliance on teleworking.

**Supplementary Information:**

The online version contains supplementary material available at 10.1186/s12889-023-17037-0.

## Background

The consequences of the COVID-19 pandemic and of the health restrictions aimed at containing it on the mental health of employed populations have been amply documented. Although children and adolescents have been identified as being particularly vulnerable to the consequences of this pandemic [1], the mental health impact on these populations has been significantly less documented than that on the adult population [2–4].

The pandemic context, including the juxtaposition of health restrictions, has proven to be fertile ground for the emergence or accentuation of work and work-family conflict (WFC) stressors, particularly among populations in situations of vulnerability [5]. Furthermore, these stressors are liable to affect not only workers’ mental health, but also that of their children, although this latter relationship requires further examination [6]. Indeed, while parental mental health has been identified as a potential mediator in this relationship, very few studies have tested this hypothesis, and almost all research on this topic was conducted prior to the COVID-19 pandemic. In response to the pressing need to document this issue, this study aims (1) to test associations between work stressors, WFC, parents’ mental health and parental concern for their children’s mental health (PCCMH), and (2) to assess whether these associations differ as a function of teleworking.

### COVID-19, workplace stressors and work-family conflict

The public health interventions deployed to curb the COVID-19 pandemic, including lockdowns, the imposition of telework, and the closure of nonessential services, schools and daycares, have highlighted the substantial contribution of social determinants, including living and working conditions, to the etiology of mental health [7, 8]. A robust association between work-related stressors and workers’ mental health had already been demonstrated before the onset of the COVID-19 pandemic [9, 10]. In populations most affected by the COVID-19 pandemic and the interventions deployed to contain it, studies have documented severe increases in work-related stressors such as work schedule adjustment, job insecurity and work overload [5, 11, 12]. Accordingly, this intensification of stressors among large swaths of the population is thought to have contributed to the increased prevalence of depressive symptoms observed since the beginning of the health crisis [11].

The COVID-19 pandemic has also highlighted deeply rooted issues such as WFC. Indeed, this stressor has long been identified as a major determinant of mental health and has now been recognized as a significant public health issue [13, 14]. WFC is defined as an inter-role conflict in which the pressures of work and family are mutually incompatible to varying degrees [15]. WFC is bidirectional in nature: work can interfere with family (WIF), and family can interfere with work (FIW). Although these two concepts are related, research shows that they represent distinct constructs, each with its own cause and consequence [13, 16]. While family factors such as tensions with children would be more of an antecedent to family-to-work conflict, work-based factors such as overload would contribute more to work-to-family conflict.

The experience of WFC during the pandemic remains to be fully elucidated as some studies report conflicting findings [5, 17, 18]. Furthermore, studies that have documented the impact of WFC on depressive symptoms during the pandemic remain uncommon. Some existing studies conducted with specific populations during the COVID-19 pandemic nevertheless suggest that both directions of WFC are associated with increases in depressive symptoms [17, 19].

### Parent’s stressors and children’s mental health

In addition to contributing to the deterioration of mental health among working parents, work stressors and WFC may play a significant role in the mental health of their children. Indeed, the proliferation of stress goes beyond the social roles of a single individual [20, 21]. While the concept of spillover refers to the situation where the stressors in one role generate tensions in another role (e.g., WFC), the concept of crossover has been put forward to refer to the transmission of stress within an interdependent role set. According to this perspective, stress experienced at work may spill over to other family members, such as spouses or children [22, 23]. In support of this hypothesis, a systematic review suggests a significant relationship between WFC and spousal psychological distress [24]. This stress contagion would also be present among the parent‒child dyad. However, this perspective has been significantly less documented than that among the adult population, particularly with respect to work stressors and WFC.

Nevertheless, there is some evidence suggesting that the parents’ work environment affects children’s mental health. Specifically, studies show that irregular work schedules are associated with externalized problems as well as internalized problems such as depressive or anxiety symptoms among children and adolescents [25, 26]. Johnson’s [27] study showed that the number of hours worked was related to internalized and externalized problems. A literature review by Mauno [28] provided an overview of the impact of job insecurity on a range of family consequences. Their review supports that job insecurity is linked to a range of outcomes for children, including reduced well-being. In a pandemic context, it has been suggested that the stress experienced by parents may also impair the ability to adequately meet the demands of parenting roles and cause adverse childhood experiences (e.g., abuse, neglect, violence) [29]. However, the associations between work-related stressors during the COVID-19 pandemic with children’s mental health remains to be explored.

Regarding WFC, studies have shown that it is associated with many manifestations among children, including mental health [6, 30–32], internalized problems [33–36] emotional problems [37], externalized problems [38], and aggression [39]. However, other studies find no significant direct relationship [40–43]. These mixed findings invite us to further explore the processes involved in these relationships.

According to the stress contagion perspective, WFC and work-related stressors do not intervene directly on children’s mental health but rather indirectly through, for example, parents’ mental health problems. Some studies have indeed shown that parents’ mental health mediates the relationship between WFC and their children’s mental health [6, 30, 31]. However, most of these studies were conducted in Australia and need to be replicated in other settings. Furthermore, although the quality of the family environment or the stress experienced by parents are listed as contributing factors, few studies have documented the impact of WFC during the COVID-19 pandemic. Among the few such studies, Wang et al. [32] found that, in a population of Chinese children aged between 12 and 15 years, parental WFC is positively related to children’s mental health difficulties. However, rather than a direct association, the results suggest an indirect relationship through parents’ mental health difficulties [32].

Although by 2022 most public health restrictions were lifted in Western countries, teleworking remained widespread in many countries [44]. If the transition was already underway, the pandemic context served as a catalyst to accelerate the transformation of this way of working. The relationship between stressors and the mental health of parents and their children could differ substantially depending on this possibility of teleworking. However, the consequence of teleworking remains equivocal. Although some beneficial aspects have been identified, many studies have shown that teleworking could be associated with difficulties in establishing boundaries between work and family, loneliness, isolation, irritability and mental health symptoms such as depressive symptoms [45–47]. This situation may be more conducive to the contagion of stress within the family when the boundaries between roles are ambiguous.

The modulation of stressors during the pandemic and the progression of telework could have long-term effects on children, as the imprint of early childhood experiences on their development and health is well documented [8]. The lack of data on the consequences of work stressors and WFC on the mental health of parents and children during the pandemic raises serious doubts as to the capacity to intervene efficiently and mitigate the possible deleterious effects in the longer term.

**Fig. 1:**
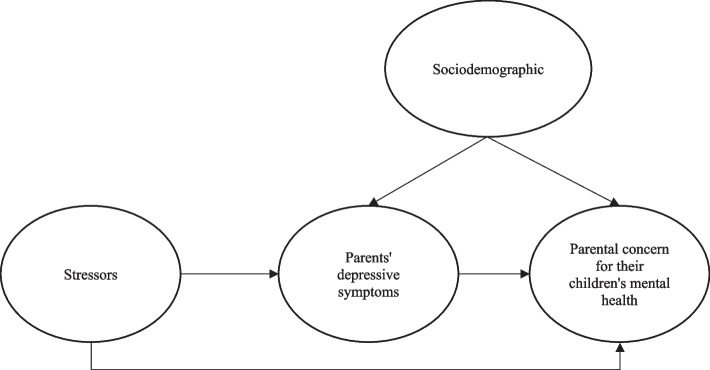
Conceptual model

Given these considerations, this study proposes to test the model presented in Fig. 1. According to this model, work stressors and WFC are positively associated with parents’ depressive symptoms. The parents’ depressive symptoms are positively linked with increases in parental concern for their children’s mental health (PCCMH). Finally, work stressors and WFC are also indirectly associated with more PCCMH through parents’ depressive symptoms. This model will then be stratified based on whether an employed parent does at least some of his or her work at home.

## Methods

This study used data from the online Health and Employment Survey of the general Quebec population conducted between April 27 and July 3, 2022. The survey solicited 4,321 members of a cohort initiated during the COVID-19 pandemic, with the addition of an oversample of 1,250 parents, to reach about 2,000 parents with children under 18 [7]. A quota sampling method for age, sex categories and education was used to ensure representativity from the general population of Quebec. In addition to sociodemographic, socioeconomic and health-related quality of life questions, this survey included questions related to work stressors, WFC and mental health of children in all age groups. Participants were invited to complete the survey through the services of Dynata Inc., and data were hosted on an internal server at our university. Of a total of 3,010 adults who agreed to participate, 1,274 were parents with at least one child under the age of 18. Of these, 878 were employed at the time of the survey. Following the removal of cases with missing data, this study includes a sample of 780 employed parents. Analysis of missing data shows that those who didn’t answer certain items worked significantly fewer irregular hours and were older (mean = 38.80 vs. 41.84). This project received approval from the Research Ethics Board of the centre intégré universitaire de santé et de services sociaux de l'Estrie (Centre hospitalier universitaire de Sherbrooke) and the Research Ethics Board of the faculty of medicine and health science (McGill University).

### Variables

#### Parental concern for their children’s mental health

Parental concern for their children’s mental health was determined from two items derived from Statistics Canada [48, 49]. Parents were asked the following question: Due to the COVID-19 pandemic, how concerned are you about the following for your child or children. One item dealt with managing stress levels and the other with managing anxiety and emotions. Responses for each item ranged from ‘1—Not at all’ to ‘4 – Extremely’. Children’s stress and anxiety management items were summed (Cronbach’s alpha = 0.85).

#### Parents’ depressive symptoms

Parents’ depressive symptoms were obtained from questions from the DASS-21 [50]. This scale includes three subscales dealing with stress, anxiety, and depressive symptoms. The 7 questions on depressive symptoms were summed (e.g., I felt down and sad) (Cronbach’s alpha = 0.94). Possible responses range from ‘0—does not apply to me at all’ to ‘3—applies to me entirely or most of the time.’

#### Work stressors

Some of the work stressors were derived from the short version of the effort-reward imbalance questionnaire [51]. The work demands were constructed from 3 items (e.g., I am constantly pressed for time because of a heavy workload) (Cronbach’s alpha = 0.75). Job insecurity was measured by combining 2 items (e.g., My job security is threatened) (Cronbach’s alpha = 0.78), and esteem received at work was measured by 2 items (e.g., Considering all my efforts, I receive the respect and esteem I deserve for my work) (Cronbach’s alpha = 0.69). The scores for the latter were reversed to represent a lower level of esteem received at work. Hours worked correspond to the number of hours worked in the last 7 days. Teleworking is a dichotomous variable (0 = No, 1 = Yes) based on responses to the following question: “Not counting overtime, do you usually do some of your paid work at home?”.

#### Work-family conflict

WFC was measured using the scale proposed by Netemeyer, Boles and McMurrian [52]. Five items are used for work-family interference (e.g., The demands of my work interfere with my home and family life) (Cronbach’s alpha = 0.92), and five other items measure family-work interference (The demands of my family or spouse/partner interfere with work-related activities) (Cronbach’s alpha = 0.88). Responses are based on a Likert scale ranging from ‘1 – Strongly Disagree’ to ‘7 – Strongly Agree’. The increased difficulties in work-family balance is based on the following question: Compared to the initial quarantine period (March–May 2020), have the difficulties of balancing your work and family life changed? Responses were on a Likert scale (‘1 – Difficulties in balancing have increased significantly’ to ‘5 – Difficulties in balancing have decreased significantly’). Responses were recoded to construct a dichotomous variable that identifies respondents whose work-family balance difficulties have increased.

### Covariates

Parental concern for their children’s mental health and parents’ depressive symptoms were controlled for citizenship status (0 = Immigrant; 1 = Canadian citizenship) and whether one child was under 5 years of age (0 = No; 1 = Yes). The younger child’s physical health status was obtained from the following question: Due to the COVID-19 pandemic, how concerned are you about the following factors for your child: physical health. The variable was transformed into a binary where 0 corresponds to ‘not at all or somewhat concerned’ and 1 corresponds to ‘very or extremely concerned’. The model also controls for parents’ sex categories (0 = Male; 1 = Female) and parents’ age in the case of parents’ depressive symptoms.

### Analyses

Statistical analyses were conducted using STATA S.E. 17.0 (Stata Corp., TX, USA). A path analysis was performed to test the model. This method is adapted to test multiple direct and indirect associations of a model that simultaneously considers all the relationships. This method also allows us to obtain fit indices of the model with respect to the data. In this study, we used the Root mean square error of approximation (RMSEA; good fit below 0.06), the comparative fit index (CFI; good fit above 0.95) and the Standardized Root Mean Squared Residual (SRMR; Good fit below 0.08) as indicators of the model’s fit to the data [53]. The model was estimated with 1,000 bootstrap replications. The standardized coefficients of the indirect relationships were reported and were calculated from the STATA estat teffects command following the model estimates [54]. We also obtained confidence intervals with the corrected bootstrap bias method with 1,000 replications to assess whether indirect effects are significant. The indirect association between X (stressors and socio-demographic variables) and Y (PCCMH) through M (parents’ depressive symptoms) is represented as ind = a*b where *a* corresponds to the regression coefficient between the variable X and M while *b* represents the regression coefficient between the variable M and the variable Y. Preliminary tests (correlation table, histogram of residuals, residual plot vs fitted, Kernel density graph, Q-Q plot), were carried out to ensure the linear and normal distribution of the residuals in multivariate regressions, as well as the absence of multicollinearity and heteroskedasticity.

The same model was then tested but stratified by teleworking status. The coefficients were then compared according to the formula proposed by [55] to test whether there is a significant difference between these two groups.

## Results

Descriptive analyses are presented in Table 1. Considering the unexpected very strong correlation between WIF and FIW, the latter was removed from the model. The results of the direct relationships are presented in Table 2. The indicators suggest that the model fits well with the data (RMSEA = 0.043; CFI = 0.996; SRMR = 0.006). Analyses show that work-to-family interference, increased difficulties in work-family balance, having an irregular work schedule, lower levels of esteem received at work, and greater job insecurity were associated with higher levels of parents’ depressive symptoms. Conversely, being in a relationship, age and citizenship status were associated with fewer depressive symptoms. The results in Table 2 also show that more parents’ depressive symptoms, WIF, increased difficulties in work-family balance, perceived children’s physical health problems and demands at work are associated with more PCCMH. Low esteem received from work and having a child under 5 years of age were associated with less PCCMH. Sensitivity analyses show that the results remain similar after controlling for family income and considering other methods of estimating standard errors without improving the model (results available on request).

**Table 1:** Descriptive analysis

	Mean/%	SD	Min–Max
Parental concern for their children’s mental health	4.81	1.77	2–8
Parents’ depressive symptoms	9.09	6.87	0–21
Couple	78.59%		
Women	50.38%		
Age (y)	38.81	8.53	17–64
Work-to-family conflict	17.75	8.33	5–35
Increased difficulties in work-family balance	52.82%		
Teleworking	60.12%		
Working hours per week	32.36	14.51	0–89
Irregular schedule	54.87%		
Low esteem received at work	4.26		
Demands	7.65	2.40	6–24
Job insecurity	4.67	1.97	2–8
Canadian citizenship	67.05%		
Child under 5 years	31.92%		
Child physical problems	32.82%

**Table 2:** Path analysis results

	Parents’ depressive symptoms		Parental concern for their children’s mental health	
Parents’ depressive symptoms			0.06^***^	[0.041;0.088]
Couple	-1.03^*^	[-1.907;-0.154]		
Women	-0.41	[-1.083;0.270]		
Age (y)	-0.06^**^	[-0.098;-0.015]		
Work-to-family conflict	0.21^***^	[0.158;0.266]	0.04^***^	[0.018;0.055]
Increased difficulties in work-family balance	2.16^***^	[1.277;3.036]	0.32^**^	[0.093;0.568]
Teleworking	0.82^*^	[0.008;1.629]	0.05	[-0.199;0.296]
Working hours per week	0.00	[-0.017;0.026]	-0.00	[-0.008;0.004]
Irregular schedule	1.47^**^	[0.541;2.407]	-0.05	[-0.317;0.223]
Low esteem received at work	0.27^*^	[0.055;0.490]	-0.11^***^	[-0.166;-0.048]
Demands	-0.06	[-0.245;0.117]	0.07^*^	[0.016;0.122]
Job insecurity	0.98^***^	[0.720;1.242]	0.05	[-0.022;0.119]
Canadian citizenship	-2.48^***^	[-3.549;-1.406]	-0.13	[-0.413;0.146]
Child under 5 years	-0.02	[-0.803;0.758]	-0.27^*^	[-0.485;-0.057]
Child physical problems	-0.33	[-1.134;0.471]	1.12^***^	[0.877;1.359]
RMSEA		0.043		
CFI		0.996		
SRMR		0.006		

95% confidence intervals in brackets; ^*^ *p* < 0.05, ^**^ *p* < 0.01, ^***^ *p* < 0.001

**Fig. 2:**
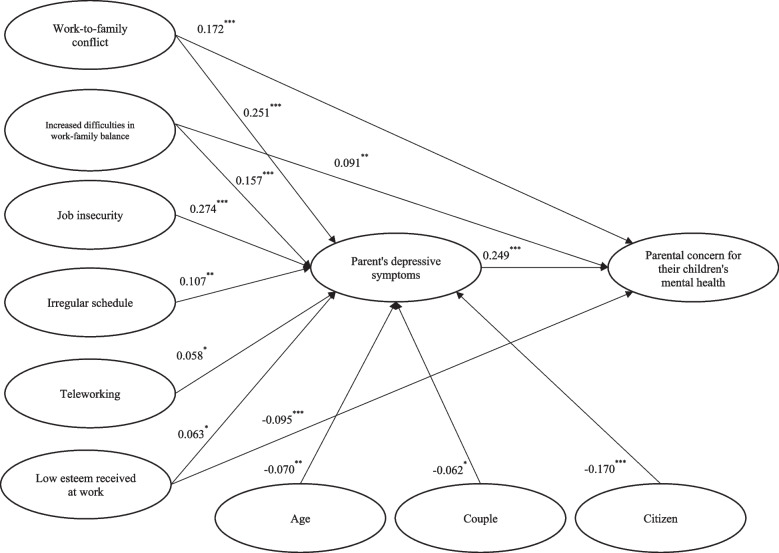
Standardized direct association between stressors, parents’ depressive symptoms and parental concern for their children’s mental health. Only statistically significant coefficients are displayed (*p < 0.05 ** *p* < 0.01 *** *p* < 0.001)

The results of the indirect relationships in the model are presented in Table 3. The latter shows that WIF, increased difficulties in work-family balance, irregular schedules, low esteem received from work and job insecurity were indirectly associated with an increase in parental concern for their children’s mental health through an increase in parents’ depressive symptoms. On the other hand, being in a couple, being older and being a Canadian citizen were indirectly related to a decrease in PCCMH through fewer parents’ depressive symptoms. Indirect associations that are significant are presented in Fig. 2.

**Table 3:** Standardized indirect associations of the path analysis with bias-corrected bootstrap confidence interval (1,000 replications)

	Parental concern for their children’s mental health	
	Indirect associations	Bias-corrected confidence interval
Work-to-family conflict	0.062^***^	[0.033;0.092]
Couple	-0.015^*^	[-0.029;-0.002]
Women	-0.007	[-0.021;0.006]
Age (y)	-0.017^*^	[-0.034;-0.001]
Increased difficulties in work-family balance	0.039^***^	[0.018;0.060]
Working hours per week	0.002	[-0.009;0.014]
Child physical problems	-0.006	[-0.020;0.009]
Irregular schedule	0.027^**^	[0.009;0.044]
Low esteem received at work	0.016^*^	[0.002;0.029]
Demands	-0.006	[-0.021;0.010]
Job insecurity	0.068^***^	[0.038;0.098]
Canadian citizenship	-0.042^**^	[-0.068;-0.017]
Child under 5 years	-0.000	[-0.014;0.013]

95% confidence intervals in brackets, ^*^p < 0.05, ^**^ *p* < 0.01, ^***^ *p* < 0.001

The analyses (in appendix) show that stressors and outcomes differ depending on the teleworking situation. Those who telework have significantly more parental concern for the child’s mental health, more depressive symptoms, work-to-family conflict, increased difficulties in work-family balance, irregular schedule, demands, insecurity and less working hours per week. Table 4 contrasts those who report teleworking with those who do not. Several factors were associated with an increase in PCCMH in both groups, including depressive symptoms, WIF, and child’s physical health problems. Low esteem received from work was negatively related to PCCMH in both groups. However, while demands at work were associated with more PCCMH, these associations were negative in the case of the number of hours worked and a child under 5 years of age among those who do not telework only.

**Table 4:** Path analysis results stratified by teleworking status

	Parents’ depressive symptoms		Parental concern for their children’s mental health	
	No telework	Telework	No telework	Telework
Parents’ depressive symptoms			0.063^**^	0.059^***^
Couple	-2.356^***^	0.005^†††^		
Women	-0.460	-0.181		
Age (y)	-0.072^*^	-0.049		
Work-to-family conflict	0.202^***^	0.229^***^	0.042^*^	0.045^***^
Increased difficulties in work-family balance	1.133	2.166^***^	0.325	0.305
Working hours per week	-0.035	0.016	-0.028^**^	0.004^†^
Irregular schedule	-0.377	2.987^***†††^	-0.216	0.043
Low esteem received at work	0.217	0.158	-0.120^*^	-0.091^*^
Demands	0.212	-0.227^†^	0.105^**^	0.039
Job insecurity	1.051^***^	0.859^***^	-0.016	0.075
Canadian citizenship	0.880	-2.643^***†^	-0.240	-0.043
Child under 5 years	-0.615	0.090	-0.520^**^	-0.122
Child physical problems	0.240	-0.603	1.055^***^	1.145^***^

Within group significance ^*^p < 0.05; ^**^p < 0.01; ^***^p < 0.001. Between groups difference significance (no telework vs telework) ^†^*p* < 0.05; ^††^*p* < 0.01, ^†††^*p* < 0.001

The picture is much more contrasted for depressive symptoms. In fact, only WIF was linked with more depressive symptoms while low esteem received from work was linked to less depressive symptoms for both teleworkers and non-teleworkers. For those who were not teleworking, being in a couple and age were negatively related to depressive symptoms. For those who telework, increased difficulties in work-family balance and irregular work schedules were linked to more depressive symptoms, while being a Canadian citizen was negatively linked to depressive symptoms.

Table 5 presents the indirect association of the model stratified by teleworking status. Among those who do not telework, WIF and job insecurity were indirectly linked to more PCCMH through more depressive symptoms. For those who telework, WIF, increased difficulties in work-family balance, irregular hours and job insecurity were indirectly linked to more PCCMH through more depressive symptoms. Moreover, being in a couple, Canadian citizenship and age were indirectly associated with less PCCMH through fewer depressive symptoms.

**Table 5:** Standardized indirect associations of the stratified path analysis model with bias-corrected bootstrap confidence interval (1,000 replications)

	No telework (*n* = 311)		Telework (*n* = 469)	
Couple	-0.037^**†^	[-0.065;-0.010]	0.000	[-0.015;0.015]
Women	-0.008	[-0.027;0.011]	-0.003	[-0.019;0.012]
Age (y)	-0.023	[-0.050;0.004]	-0.014	[-0.034;0.006]
Work-to-family conflict	0.060^*^	[0.011;0.109]	0.064^***^	[0.027;0.101]
Increased difficulties in work-family balance	0.019	[-0.008;0.045]	0.036^**^	[0.013;0.059]
Working hours per week	-0.012	[-0.035;0.011]	0.010	[-0.005;0.024]
Irregular schedule	-0.006^††^	[-0.027;0.015]	0.048^**^	[0.018;0.078]
Low esteem received at work	0.012	[-0.009;0.033]	0.009	[-0.007;0.025]
Demands	0.018^†^	[-0.006;0.042]	-0.019	[-0.043;0.004]
Job insecurity	0.060^**^	[0.017;0.103]	0.059^**^	[0.024;0.094]
Canadian citizenship	0.008^††^	[-0.016;0.032]	-0.047^**^	[-0.078;-0.015]
Child under 5 years	-0.010	[-0.032;0.013]	0.002	[-0.015;0.018]
Child physical problems	0.004	[-0.017;0.024]	-0.010	[-0.028;0.007]

95% confidence intervals in brackets. Within group significance ^*^p < 0.05; ^**^p < 0.01; ^***^p < 0.001. Between groups difference significance (no telework vs telework) ^†^*p* < 0.05; ^††^ *p* < 0.01, ^†††^ *p* < 0.001

## Discussion

The purpose of this study was to test a model of the relationship between work stressors, WFC, parental depressive symptoms, and parental concern for their children’s mental health. This model was tested for indirect relationships between stressors and parental concern for their children’s mental health. This model was also contrasted according to teleworking status during the COVID-19 pandemic. The stress contagion hypothesis is partially supported considering that many indirect associations were observed, while direct associations remained significant between stressors and the PCCMH. Although the measure used in this study differs from that in other studies, the results were largely consistent with what was found with internalized problems and other mental health measures among children reported by the parents. Nevertheless, this research sheds singular light on the link between the work environment and mental health during the COVID-19 pandemic.

First, work stressors were related to PCCMH but involved distinct processes. While work demands were positively related to PCCMH, the relationships were more indirect for job insecurity and irregular work schedules during COVID-19. Low esteem received from work was the only stressor that was both directly and indirectly related to PCCMH through parents’ depressive symptoms. The indirect relationships of irregular hours, low esteem received from work and job insecurity were consistent with the stress contagion hypothesis. The labor market was profoundly disrupted during COVID-19, and a significant proportion of the population became unemployed or had their schedules modified [56]. This context was conducive to the emergence or amplification of job insecurity, low perceived work esteem, and irregular work schedules [11]. It is possible that the very nature of these work stressors can more easily affect the family sphere, considering, for example, that job insecurity can fuel financial insecurity and familial tensions. Irregular work schedules may also require significant adaptations from the family, in addition to being associated with more fatigue and irritability among workers and affecting human relations [57]. While studies have supported the influence of these stressors on the family environment, this study is the first to our knowledge to document potential relationships with PCCMH through parents’ depressive symptoms since the onset of the COVID-19 pandemic.

Second, the level of WIF as well as the increased difficulties in work-family balance were directly associated with the PCCMH and indirectly associated through the increase in depressive symptoms. This is also consistent with the stress process perspective given the proliferation of stress across the interdependent social roles of one or more individuals [21]. On the one hand, our study reaffirms the importance of considering WFC in research on the psychosocial determinants of worker health. On the other hand, it calls for serious consideration of the potential effects of WFC on children’s mental health during a pandemic. If initiatives are taken in this direction, they should be considered by their jurisdiction to allow prompt intervention in an informed manner on this issue.

Third, comparing the pattern between those who telework and those who do not revealed another nuanced story. First, we found that the increased difficulties in work-family balance was indirectly related to PCCMH, but only in the teleworking group. Since the direct relationship was not significant, this result supports a total mediation that is more consistent with the stress contagion hypothesis. This is also consistent with studies showing that teleworking may be associated with more blurred spatial and temporal boundaries between work and family, which may contribute to spread negative emotions from work more easily [45, 47]. This has important implications for research on youth mental health. As we see a marked growth in telework that appears to be persisting beyond the acute phase of the pandemic, it is important to further document how the quality of the conditions under which teleworking occurs can influence children’s mental health and well-being. Although beyond the scope of this study, it is also possible that favorable teleworking conditions can contribute to work-family enrichment and, consequently, to the well-being of parents and children [6, 45, 47].

Furthermore, while work hours were not related to PCCMH, stratified models showed that the latter was negatively associated among those who do not telework. This negative relationship in this group may be related to income and job security. Indeed, a significant number of hours could act as a protective factor in a period of instability such as a pandemic. Another case refers to work demands, the direct association of which was only observed for those who were not teleworking. Although this does not necessarily refute stress contagion, it is possible that aspects other than depressive symptoms may mediate this relationship. For example, it is possible that high work demands could negatively affect parental involvement or the quality of relationships with children, with deleterious effects on children’s mental health. Although these hypotheses could not be tested in this study, these relationships deserve to be further explored in the context of labor disruptions such as those brought on by the COVID-19 pandemic.

This study is not without limitations. First, the measure used as a proxy to capture children’s mental health, though it is derived from that used by Statistics Canada, is not widely used or validated in the literature. Although other studies will have to be replicated with validated scales, the results are largely consistent with what was found in the literature prior to COVID-19. Second, since questions on WFC and perceived parental concern for their children’s mental health were added in the last wave of the survey with an oversampling of parents, it is not possible to conduct longitudinal analyses and our results are liable to reverse causality. However, previous studies have validated the causal direction of the relationships between stressors and mental health, lending external validity to this study [9, 10, 30, 31, 58]. Although beyond the scope of this study, the sample size also limits the possibilities of testing these models stratified by social demographic and socioeconomic groups to identify inequalities that may be at work. Nevertheless, we controlled for some of them, such as age, sex categories, and Canadian citizenship status.

## Conclusion

This study contributes to knowledge regarding the determinants of children’s mental health during the COVID-19 pandemic by testing a comprehensive model integrating a multitude of stressors deriving from work and work-family interfaces and by identifying the specific processes involved. Notably, our results invite serious consideration of the long-term impact of WFC, job insecurity, irregular work schedules, teleworking, and low esteem received at work on the mental health of children following the COVID-19 pandemic. This is particularly relevant at a time when data on this topic are sorely lacking yet necessary to inform decision makers and guide actions. Considering the massive use of teleworking and the potentially deleterious effects on family members, efforts should be made by jurisdictions and organizations to prevent work-related psychosocial risks.

### Supplementary Information

**Additional file 1.**

## Data Availability

The datasets used and/or analysed during the current study are available from the corresponding author on reasonable request.

## Abbreviations

FIW: Family interfering with work
PCCMH: Parental concern for their children’s mental health
WFC: Work-family conflict
WIF: Work interfering with family
