# Supplementary material for: Work stressors, work-family conflict, parents’ depressive symptoms and perceived parental concern for their children’s mental health during COVID-19 in Canada: a cross-sectional analysis
Source: BMC Public Health. 2023 Nov 7;23:2181. doi: 10.1186/s12889-023-17037-0 (PMC10629015; doi:10.1186/s12889-023-17037-0)
Supplement: Supplementary file 1 — Additional file 1. [file 12889_2023_17037_MOESM1_ESM.docx]

Supplementary table S1. Difference in stressors and outcomes according to

teleworking status

|  | No teleworking | Teleworking |
| --- | --- | --- |
|  | Mean/% | Mean/% |
| Parental concern for their children's mental health | 4.26^***^ | 5.18 |
| Parents' depressive symptoms | 5.94^***^ | 11.18 |
| Work-to-family conflict | 15.64^***^ | 19.14 |
| Increased difficulties in work-family balance | 30.55%^***^ | 67.59% |
| Working hours per week | 36.02^***^ | 29.92 |
| Irregular schedule | 30.87%^***^ | 70.79% |
| Low esteem | 4.24 | 4.27 |
| Demands | 15.16^***^ | 16.17 |
| Job insecurity | 3.91^***^ | 5.17 |

Note : T test for continuous variable and chi^2^ for binary variable

^*^ *p* < 0.05, ^**^ *p* < 0.01, ^***^ *p* < 0.001
